# Who Masturbates? It Depends: Predictors of Masturbation by Gender and Partnership of Older Adults

**DOI:** 10.1093/geroni/igaa057.1006

**Published:** 2020-12-16

**Authors:** Patricia Barrett, Elisabeth Burgess

**Affiliations:** 1 Georgia State University, Marietta, Georgia, United States; 2 Georgia State University, Atlanta, Georgia, United States

## Abstract

Studies on masturbation often exclude older adults. While the frequency of masturbation is known to decline over the life course, research has neglected masturbation in older adulthood. In the adult population, the connection between partnered sex and masturbation has been well established; this relationship is mediated by individual’s contentment with their sex life. Furthermore, the sex/masturbation relationship is distinct by gender. This study seeks to understand how these relationships exist and influence masturbation among older adults. Using data from the third wave of the National Social Life, Health and Aging Project (NSHAP) (N=2,006), we completed logistic regressions to examine how sexual thoughts, frequency and contentment with partnered sex predict older adult’s likelihood of masturbating. Preliminary analyses, controlling for partnership status, gender, age, race, mental and physical health, indicate that frequent sexual thoughts (OR 4.52) and discontentment with sex frequency (OR 1.46), rather than frequent partnered sex, are significantly associated with an increased likelihood of masturbating in the year prior. Employing separate regressions for gender, bothersome sexual dysfunctions and higher depression scores were significantly associated with increased odds of masturbating for women, but not men. For partnered older adults, only those who reported relationships that were both physically and emotionally satisfying were less likely to masturbate (OR 0.54). To our knowledge, this is the first study that considers masturbation as a sexual behavior with distinct predictors by gender and partnership status. We conclude by discussing the implications of nuanced and varying sexual desires and behaviors among the older adult population.

